# Changing Neighborhoods and Residents’ Health Perceptions: The Heart Healthy Hoods Qualitative Study

**DOI:** 10.3390/ijerph15081617

**Published:** 2018-07-31

**Authors:** Paloma Conde, Marta Gutiérrez, María Sandín, Julia Díez, Luisa N. Borrell, Jesús Rivera-Navarro, Manuel Franco

**Affiliations:** 1 Social and Cardiovascular Epidemiology Research Group, School of Medicine, University of Alcalá, Alcalá de Henares, 28871 Madrid, Spain; p.conde@uah.es (P.C.); magusa@usal.es (M.G.); maria.sandin@uah.es (M.S.); julia.diez@uah.es (J.D.); jrivera@usal.es (J.R.-N.); 2 Department of Sociology and Communication, University of Salamanca, 37007 Salamanca, Spain; 3 Department of Epidemiology and Biostatistics, Graduate School of Public Health and Health Policy, City University of New York (CUNY), New York, NY 10027, USA; luisa.borrell@sph.cuny.edu; 4 Department of Epidemiology, Johns Hopkins Bloomberg School of Public Health, Baltimore, MD 21205, USA

**Keywords:** urban health, health perceptions, qualitative research, neighborhoods, social change, Spain

## Abstract

Cities, and therefore neighborhoods, are under constant change. Neighborhood changes may affect residents’ health in multiple ways. The Heart Healthy Hoods (HHH) project studies the association between neighborhood and residents’ health. Focusing on a middle–low-socioeconomic neighborhood in Madrid (Spain), our aim was to describe qualitatively its residents’ perceptions on the urban changes and their impacts on health. We designed a qualitative study using 16 semi-structured interviews including adult residents and professionals living or working in the area. Firstly, we described the perceived main social and neighborhood changes. Secondly, we studied how these neighborhood changes connected to residents’ health perceptions. Perceived major social changes were new demographic composition, new socio–cultural values and economic changes. Residents’ negative health perceptions were the reduction of social relationships, increase of stress and labor precariousness. Positive health perceptions were the creation of supportive links, assimilation of self-care activities and the change in traditional roles. Neighborhood changes yielded both negative and positive effects on residents’ health. These effects would be the result of the interrelation of different elements such as the existence or absence of social ties, family responsibilities, time availability, economic resources and access and awareness to health-promoting programs. These qualitative research results provide important insight into crafting urban health policies that may ultimately improve health outcomes in communities undergoing change.

## 1. Introduction

Cities are dynamic structures in constant change [1]. Lefebvre highlighted the ability of the city to transform the environment: “*in urban space something is always happening. Relations change. Differences and contrasts can result in conflict, or are attenuated, erode or corrode*” [2]. Since Lefebvre’s study, cities have undergone many changes amid the intertwining relationship between individuals and structure. According to Giddens’ Theory of Structuration, individuals living in cities as urban agents not only accept the structure elements (rules and resources) surrounding them, but also modify the urban context constantly with their practices [3]. Inclusion of variables such as age, gender, ethnicity or class allowed us to explore how different population groups cope with urban changes [4].

Together, globalization, impact of technologies, and displacement of industrial production to new geographic areas shape the new urban context as a whole [5]. Alongside these and importantly, the impact of international migration and the increase of social inequality affect the current urban settings by adding diversity and complexity of its internal dynamics [6]. Parallel to this macro perspective, urban analysis calls attention to the micro every-day life because urban space cannot be distinguished from the “social” environment where people interact [7]. Therefore, neighborhoods are the closest spatial and social framework to study its resources along with its forms of sociability.

Alongside the social individual interactions within cities and urban spaces, these contexts affect the distribution of health and disease because neighborhoods provide networks of social relationships with implications for health [8]. For instance, previous studies focusing on features within a neighborhood suggest that a neighborhood makes its residents have better or worse health [9,10], or that there are possible structural changes that may help improve residents’ health [11]. Moreover, several studies have explored how neighborhood environment shapes physical activity [12,13,14], dietary patterns [15,16] or health perceptions [17,18,19]. Within this framework, we argue that changes to neighborhoods affect new forms of social desirability for residents’ perception of health by affecting individuals’ everyday beliefs, behaviors and practices related to health, such as social relationships and support, health behaviors and other health-related practices such as healthcare access and use. The latter may directly or indirectly impact health status of neighborhoods’ residents.

Thus, this evidence prompted us to examine how neighborhood changes may affect their residents’ health perceptions. To address this issue, we used semi-structured interviews to collect information on the daily life of a middle–low socioeconomic neighborhood in Madrid, Spain, to examine neighborhood social changes as perceived by residents and how these changes may affect residents’ health perceptions.

## 2. Materials and Methods

This qualitative study is framed within a larger study, the HHH project [20] funded by the European Research Council (ERC) aiming at characterizing the entire city of Madrid (Spain) and the cardiovascular health of its residents from 2014 through 2019 [21]. This characterization comprises the study of four health dimensions of the urban environment: the food, alcohol, tobacco and physical activity.

### Setting

Since 1960, Madrid has been developed as a modern city, being a focal point of rural immigration first, urbanism development, the incorporation of women into the workplace and recently as an international immigration host city since 2000. In 2008, the global crisis started in Spain, especially affecting the city of Madrid, with the eruption of problems such as the housing bubble, the banking crisis and a dramatic rise in unemployment. 

Aging is another common phenomenon in modern societies. In the case of Madrid, this demographic process has increased as a result of the improvement in the quality of life, higher life expectancy and a low birth rate [22].

We conducted a qualitative study in a group of neighborhoods within the district of Ciudad Lineal, in Madrid. These neighborhoods have no specific names, as they are a small part of the bigger district. Hereafter, we will name and refer to the area as Little Ciudad Lineal. Little Ciudad Lineal is close to the city center by public transportation (15 min); an area of 131,968 inhabitants (2015). From an urban point of view, the neighborhood under study is mixed, combining high-population-density areas and large buildings, with areas of old housing (four floors) that have not been remodeled. As shown in Table 1, the study area had higher proportion of immigration (foreign-born) and of population over 65 years old than Madrid City. Unemployment rates were similar for the study neighborhood and Madrid City. This urban area was already selected in a previous pilot study within the frame of the HHH project. Details on the selection method of the area are published elsewhere [21]. Briefly, the selection criteria were based on the “median neighborhood” among all neighborhoods in Madrid in terms of percentage of residents above 65 years of age or older, percentage of residents with low education, percentage residents being foreign-born, and population density. We assumed that being the typical median neighborhood in Madrid, it would be equally affected by change.

Regarding public services, the area comprises several health centers (Primary Care Health Centers and Health Promotion Centers), primary schools and Senior Centers. These centers organize activities and workshops related to the so-called healthy aging: exercising, nutrition, memory training and social network maintenance.

Between January 2014 and January 2015, we conducted 16 semi-structured interviews to adults with the objective of capturing their perceptions of social and urban changes occurring in their neighborhood and their effect on residents’ health perceptions.

For participant recruitment, we visited neighborhood associations, several educational and health centers as well as the Elderly Care Services Department, and a traditional public market. After contacting these agencies, we used the snowball approach to complete the aimed profiles.

Our study population included adult residents and workers of Little Ciudad Lineal. For participant selection, we used a stratified purposeful sampling, taking into account variables that could influence the discourse around the neighborhood (age, sex, number of years living or working in the neighborhood, country of origin, and occupation). The following inclusion criteria were used: lived or worked in the neighborhood for the last five years, and to speak Spanish. A total of 16 participants (see Table 2) were selected according to the compliance with any key variable (see Table 2, column 5) to narrate neighborhood changes. Nine participants were residents, six participants worked in the neighborhood and lived elsewhere, and one participant lived and worked in the neighborhood. Given that not every participant had the same number of years of experience in the neighborhood, we chose no specific year of reference to narrate the change.

Informed consent was obtained from all individuals included in the study. To protect participants’ identity, we used pseudonyms throughout the paper. The HHH project (ERC-2013-StG-336893) obtained ethical approval from the Ethics Research Committee of the Madrid Health Care System on 12 November 2013.

The semi-structured interview is a valuable research tool for capturing subjects’ narratives and experiences about their practices of living [24]. Previous studies suggested that neighborhood environment shapes physical activity, dietary patterns or health perceptions [12,13,14,15,16,17,18,19]. Based on these studies, we designed an interview guide (Table 3) with the purpose of collecting information on the neighborhood and the most important changes they went through, the type of activities residents do within their neighborhoods, and their opinions on how healthy (or unhealthy) their neighborhoods were, to explore how this change could impact the health perceptions or lifestyle of neighborhoods’ residents.

Interviews lasted from 45 min to 1 h, and took place at participants’ work places, residents’ homes and/or a senior center.

Three researchers (all co-authors) conducted and audiotaped the 16 interviews. Verbatims throughout the text are literal quotations extracted from participants’ narratives (cited by pseudonym and age). Consistent with previous studies [25,26], we used a qualitative descriptive method [27] to guide our qualitative analyses. Qualitative descriptive studies aim to provide a comprehensive summary of events of the day-in and day-out of those events.

Each researcher independently did several systematic transcript readings until obtaining a list of categories and subcategories. This method of analysis followed the next steps [28]: (1) categorization—categories have high abstraction level and each category could include several dimensions; (2) subcategorization—subcategories have lower abstraction level, explaining and describing deeply the main categories; and (3) a unique list of categories was obtained through inter-evaluator agreement [29].

## 3. Results

**Figure 1:**
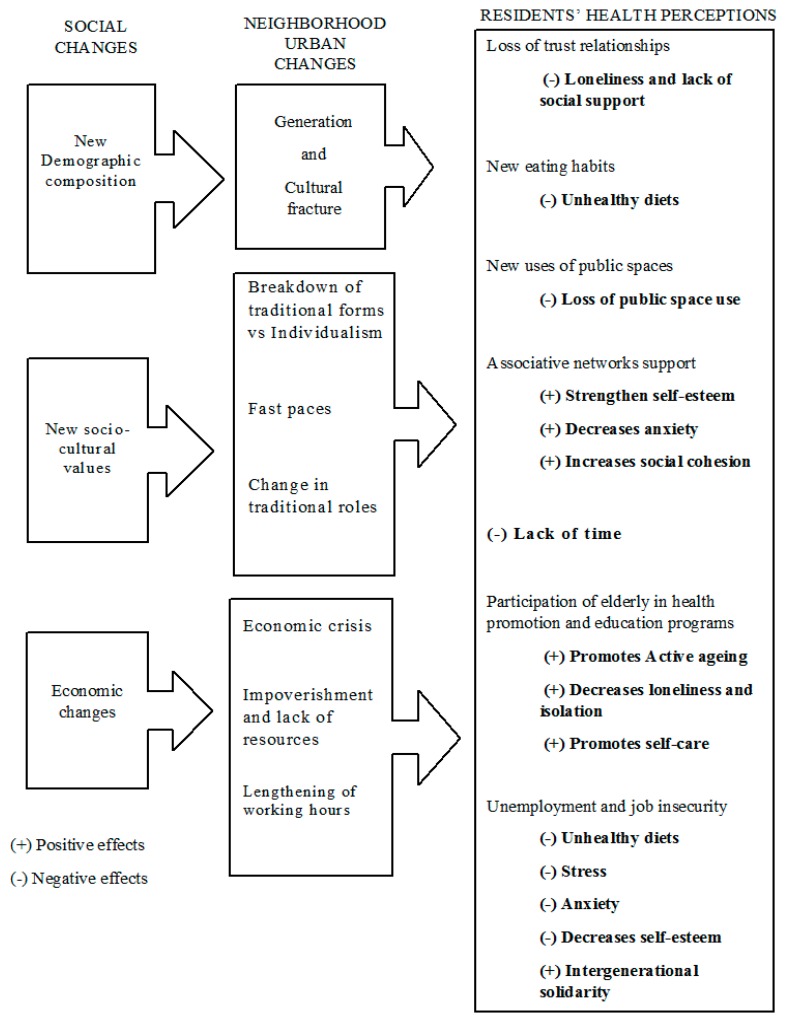
Social changes shaping neighborhood urban changes and further affecting participants’ health perceptions as explained by study participants.

Figure 1 shows the social changes shaping the neighborhood’s urban changes, further affecting participants’ perception of health in the studied neighborhood. This figure illustrates three different levels: The first level includes global components and alludes to current social changes; the second level refers to neighborhood urban changes, triggered by the social changes in the neighborhood; finally, the third level focuses on the effects on residents’ health perceptions derived from life practices determined by urban changes.

Because we found that changes may have both positive and negative effects, we used + and − symbols in front of each effect to indicate positive or negative effects.

The social changes described in the first level and identified by the study’s participants were new demographic composition, new socio–cultural values, and economic changes, which mainly focused on the economic crisis and unemployment suffered in the years before the study.

### 3.1. New Demographic Composition

Demographic composition changes mostly refer to population aging and immigration of the neighborhood. This neighborhood used to be a very homogenous population in the sixties and over recent years has become highly heterogeneous, and occasionally fragmented in terms of generational and cultural background.

The latter change has defined a sense of “we” that identifies native population, who originated the neighborhood, against “they” (“the others”). These “they” are not only immigrants but also younger people who have no previous background in it. Moreover, this fragmentation is articulated in two aspects: one has a cultural character, distinguishing natives from immigrants, and the other has a generational character, distinguishing older people from younger.

Derived from this cultural and generational fracture, we highlighted the next perceived effects on health:

Old neighbors who shaped the neighborhood keep a way of life resting on social relationships of trust and mutual support. On the contrary, younger people are not so attached to those close relations, doing most of their activities out of the neighborhood. This generational fracture seems to negatively affect health with loneliness and lack of social support (see Figure 1, (−) Loneliness and lack of social support).

“I had a neighbor who has gone with my children to the doctor, if it was necessary (…) but you no longer trust people as before. Now you have these 2nd generation neighbors who say good morning and good afternoon, but you do not connect anymore with them because they are young couples or single-parent couples.”

(Diana, 69 years)

“Here there are many people who live alone … that lady lives alone and she is 95 years, she has a sister (…) but still lives alone, and does not turn on the heating at home.”

(Julia, 38 years)

Demographic changes in relation to food consumption were also perceived and translated as the presence of new eating habits, considered as unhealthy (See Figure 1, (−) Unhealthy diets).

“Many of our elder caregivers are immigrants cooking for them, South American cuisine is very rich in carbohydrates and fat, but East European cuisine is to die from (…) it is heavy, greasy, lots of corn, lots of flour.”

(Felisa, 53 years)

Natives perceived that recently settled populations use public space more intensely, which could affect the practice of physical activity by natives in these public open spaces (see Figure 1, (−) Loss of public space use).

“You got the area where moms are with their children, and the basket courts that many times you remember in the American movies (…) immigrants have taken them (…) many times my wife tells me: “Come on, why don’t you go down with the boy to the park with the ball?” Well no, I mean, no, I don’t feel comfortable going down with my son to play there.”

(Alberto, 48 years)

“I think they (immigrants), because many of them live in small houses, spend more time in the street and use more public spaces.”

(Daniel, 62 years)

### 3.2. New Socio–Cultural Values

Participants underscored a breakdown of traditional forms towards more individualistic positions, presence of faster pace as well as changes in traditional roles.

Urban development implies the need for constant mobility for work or leisure. This accelerates pace, making it difficult to maintain the quality of social relations and fostering the emergence of individualistic values. In younger adults, it resulted in a lack of time (see Figure 1, (−) Lack of time).

“We used to enjoy more what happened to oneself, everyone used to share more or less the joys the sorrows, not anymore, nowadays we live in our houses, everything is utterly individualized, people keep to themselves, they care rather less about each other.”

(Belén, 58 years)

“As it happens to my daughters, they both work, they rush all the time and they do not have time for anything.”

(Carlos, 85 years)

In addition, the presence of instrumental associative networks, like neighborhood associations, may replace the loss of social support, responding to specific residents’ needs. Residents appreciated these actions in the face of improving their health, strengthen self-esteem, decreasing anxiety and increasing neighborhood social cohesion (see Figure 1).

“I am not only unemployed, I am coming here (neighborhood association), I stick papers, I tell my sorrows, I tell the person next to me who is in the same situation. You come here with the hope of seeing if we have achieved something. Look! How great this friend has a job now.”

(Belén, 58 years)

As part of the breakdown of traditional forms, we also found a process of role changing, in terms of both gender and age. Regarding gender, there was a great presence of older women in health education programs.

“Old women participate in every programmed activity (…) Meanwhile men are more reluctant, but it is changing, some men are starting to be more into it.”

(Irene, 57 years)

Regarding age, we found a changing role in elderly people, who are more involved not only doing new activities, many of them healthy, but also on an extension of the traditional responsibilities, such as caring of children and grandchildren.

“I usually come to the talks and workshops and on top of it social activities, but I have enough to do at home, I still have five people to feed at home, my husband, my son the squatter, then my eldest son and my granddaughter who goes to a near school. Enough is enough.”

(Diana, 69 years)

In contrast, breaking with traditional norms would legitimize changes in practices and improvements in lifestyles. Development of education health programs in daycare centers facilitates generalization of those practices in line with the so-called active aging (see Figure 1, (+) Promotes active aging). In addition, participation in these programs decreases loneliness and isolation (see Figure 1, (+) Decreases loneliness and isolation).

“I joined physical training because is very good for me, because the doctor has recommended it to me and so on but aside I also do not need to be isolated, by staying home, I need a task to get out from home to be in motion and talk to one another.”

(Julia, 38 years)

Incorporation of self-care habits (see Figure 1, (+) Promotes self-care) was perceived by participants as a higher concern in terms of healthy eating, exercising or strengthening mental health.

“People have increased the tendency to do exercise, to drink water when you tell them to drink it; it is also true that we look for healthier diets.”

(Felisa, 53 years)

### 3.3. Economic Changes

The third social change relates to economic aspects that may have shaped the life of the neighborhood in recent years. According to participants, economic crisis and the rise of unemployment led to poverty. We also collected views on high working hours. Regarding health perceptions, the first effect did relate to the reduction of household income to purchase food and fresh products (see Figure 1, (−) Unhealthy diets).

“People here are economically disadvantaged. Now there are many Chinese selling hot dogs for eighty cents.”

(Enrique, 41 years)

Long working hours and unemployment affected working people’s mental health. Perceived health was negatively affected by stress, anxiety and lack of self-esteem. (See Figure 1, (−) Stress, (−) Anxiety and (−) Decreases self-esteem.)

“Imposed overtime working days and competition and pace and things in this economic system destroy people and maybe does not make you be severely ill but it does undermine you in many aspects.”

(Daniel, 62 years)

According to participants, economic decline forced families to solve progressive impoverishment of their members. Intergenerational solidarity (see Figure 1, (+) Intergenerational solidarity) would supply care of food and other expenses, by the most stable and older generations, to younger and unstable ones.

“Nowadays there are many grandparents who are in charge of the family. Many siblings on the dole (…) An elderly patient of mine, the woman is crying (…) Oh dear I do have hamburgers because my grandson is coming to have lunch and I do not know how much is it, three or four euros, shall we eat hamburgers or not….”

(Felisa, 53 years)

“You used to have a better pension, it was a bit higher to face the situation, and if you have to help your siblings your pension lowers. Then you have to check things. You can feel it. Economic crisis did affect.”

(Alberto, 48 years)

## 4. Discussion

New demographic composition, new socio–cultural values and economic changes were the major social changes related to health in the neighborhood, according to participants.

Life changes associated with economic crisis and its political management may constitute the central axis underlying middle–low-income neighborhood daily lives. Moreover, those economic changes have the most direct impact on the perception of health. Previous studies have pointed out that impoverishment and job insecurity leave negative features on population life practices and health [30,31,32].

Regarding health, demographic changes are vital [33]. In our study, these materialized with the presence of groups—immigrants and young—which do not replicate traditional ways of life, choosing other types of social relationships and neighborhood uses, contributing to create more heterogeneous neighborhoods where traditional forms of life are maintained along with others more diverse [1].

The coexistence of traditional ways of life with more individualistic ones in the same neighborhoods prompts the fracture between older neighbors and active working adults [34], with different health effects [35,36,37]. A recent study conducted in Spain suggested that conditions associated with aging, such as widowhood or the loss of the intergenerational coexistence, increase the perception of loneliness and, in turn, increase health problems, such as depression [38]. Other studies also relate loneliness and isolation with depression and mortality [39,40]. Moreover, urban changes may affect each social group differently. Elderly are affected by loneliness and changes in relationships with neighbors, whereas young people are more affected by stress as the result of the unemployment and labor precariousness.

Other changes related to new socio–cultural values have also shown negative effects on health and wellbeing [41]. For instance, cultural changes in traditional dietary patterns have been identified as factors of body mass index (BMI) increments in an urban context [42,43]. Isolation or loss of social cohesion [44] are originated by the weakening of social relationships and social support [45]. In contrast, we highlighted positive aspects related to the release of certain social norms, fostering individual responsibility required to maintain health. Examples of positive changes affecting elderly are the value given to the responsibility on care, both self-care and care of younger generations [46], as well as the great value given to the role played by institutions and health policies in neighborhoods [47] like local health programs and actions taken around active aging [48]. Studies on leisure have detected how participation of women in the social public sphere—while maintaining responsibilities in private area—would be a positive result of socio–cultural change [49].

In short, urban transformations contain ambivalent elements as far as health is concerned; thus, for example, against the loss of social support derived from new urban life features and population heterogeneity, new links appear, some of associative character, others more informal or temporary, but also valuable resources for social protection and health. Intergenerational solidarity, as our study described, has been highlighted in South European contexts [50,51] as a response to social policy deficiencies, and could be the only positive aspect of the economic decline.

### 4.1. Limitations

Participant selection may present some level of bias. We visited public centers and associations as recruitment points. Doing so, part of the residents interviewed had some connection with social organizations (neighbor associations, daycare centers or educational centers). Probably, people with no links with those public organizations were not as well as represented. However, this is usually the case with qualitative research. Nevertheless, participants interviewed associated with social organizations had a broad and integral perspective about the neighborhood. Not every participant with links to an organization was in the same social position or reproduced similar discourse.

The study does not focus on any specific population group; the final sample is quite diverse, with higher presence of certain groups. Older residents’ narratives led us to know the history and changes of the neighborhood, but their discourse could be more represented compared to the younger population one. Still, it is honest to say that Little Ciudad Lineal, as almost every middle-class neighborhood in Madrid, is quite aged. Thus, their contribution is crucial.

We are aware of the rather small number of interviews that may not totally cover the heterogeneity of the population under study. Nevertheless, the use of a stratified purposeful sampling allowed for a complete coverage of the important concepts under study. We still think the population groups selected are on the best position to describe urban change and the relationship with the perception of health in the neighborhood.

### 4.2. Strengths

Our study addressed broadly the relationship between neighborhood changes and health. We included all aspects expressed by participants, without leading them to any concrete dimension. We also integrated at the same time institutional and public services perspectives that may play a relevant role in the area. The variety of profiles also helped gathering and integrating a large diversity of views describing the neighborhood change in a multifaceted way. This study incorporates the dynamic aspect of neighborhoods through the study of change in three dimensions: demographic, socio–cultural and economic; changes analyzed from residents’ and professionals’ points of view.

## 5. Conclusions

Our findings show that reduction of social relationships, increase of stress, and labor precariousness were perceived as health stressors, whereas the creation of associative supportive links, assimilation of self-care activities, local health programs and change in traditional roles were seen as protective health factors. Thus, neighborhood changes showed both negative and positive effects on residents’ health. These qualitative research results may provide important insight into crafting urban community health policies, especially in rapidly changing communities, directly related to community health and primary healthcare systems. Positive health outcomes may be observed from policies stimulating and protecting associative and participatory processes, as well as promoting self-care activities stemming from the healthcare system. Furthermore, policies aimed at reducing social isolation and labour precariousness, and their related stresses, may result in population health improvements. Qualitative research provides helpful and specific knowledge to appropriately intervene in our ever-changing cities and neighborhoods.

## Figures and Tables

**Table 1:** *Little Ciudad Lineal* demographic characteristics. Sociodemographic variables of the neighborhood under study.

Characteristics	Madrid City	Little Ciudad Lineal
% of foreign-born population	19.29	25.52
% population over 65 years old	20.57	23.80
% unemployment	16.32	16.10

Source: own elaboration based on Madrid City Council Data Bank [23].

**Table 2:** Characteristics of the 16 study participants.

Pseudonym	Sex	Age	Interview No.	Key Variable for Selection/Inclusion in the Study
Alberto	M	48	1	Food trader
Carlos	M	85	2	Long-time neighbor
Carmelo	M	63	3	Employed neighbor
Daniel	M	62	4	Neighborhood association activist ^1^
Enrique	M	42	5	Immigrant neighbor
Fernando	M	65	6	Long-time neighbor
Germán	M	63	7	Local politician
Ana	F	83	8	Long-time neighbor
Belén	F	58	9	Unemployed neighbor
Carmen	F	45	10	Immigrant neighbor
Diana	F	69	11	Long-time neighbor
Elena	F	41	12	Primary school teacher
Felisa	F	53	13	Primary care doctor
Gala	F	51	14	Head of Health Promotion Department
Irene	F	57	15	Head manager of Senior Care Centers
Julia	F	38	16	Recreational and Cultural Activities technician

^1^ Neighborhood association is a group of people living in the same community, legally organized to achieve common public life goals. In Spain, these associations have a long tradition of struggle to gain better living conditions in the neighborhoods.

**Table 3:** Interview topic guide.

Neighborhood description Description of the neighborhood and neighbors Geographic neighborhood boundaries Most important changes
Neighborhood uses Working place, for shopping, leisure time Time spent in the neighborhood Neighbors’ concerns Satisfaction with the neighborhood and its services
Health related to neighborhood Physical elements affecting health Practices and lifestyle related to health Proposals for neighborhood improvements related to health

## Availability of Data and Material

Data generated and analyzed during the current study are not publicly available due to participant confidentiality and anonymity conditions, but are available from the corresponding author on reasonable request.
